# Optimal strategy of in-game items with conspicuous consumption: whether to provide the grinding version?

**DOI:** 10.3389/fpsyg.2023.1259319

**Published:** 2023-12-15

**Authors:** Feng Luo, Jiaqi Chen, Tiantong Xu

**Affiliations:** Business School, Beijing Technology and Business University, Beijing, China

**Keywords:** conspicuous consumption, optimal strategy, in-game items, paid version, grinding version

## Abstract

In the virtual world, whether or not to spend money on in-game items distinguishes paying players from non-paying players. Due to the existence of conspicuous psychology, paying players will greatly increase their conspicuous utility after purchasing an item in addition to the utility of the item itself. In this case, whether providing different versions of items can bring greater revenue to the game company is a question worth investigating. In this study, two analytical models considering conspicuous intensity are developed to compare the optimal pricing strategy of the game company providing the single-version item or dual-version items. The single-version item can only be purchased, while a relatively low-quality version that can be obtained by grinding is provided in the dual-version strategy. Grinding means using time in games to get items instead of spending in games. The results suggest that it is more profitable for companies to offer dual-version items when conspicuous intensity is strong. Game companies can also adjust the time needed to acquire the grinding version item and the quality gap between the two versions to achieve greater revenue. The research contributes to providing a theoretical basis and decision support for game companies to decide whether to provide different versions of in-game items.

## 1 Introduction

The game industry has grown rapidly in recent years (Cheah et al., 2022), and transactions in virtual goods and services have grown dramatically (Ke et al., 2012). Game companies' profitable methods mainly consist of built-in advertising, software subscriptions, selling virtual goods, and other value-added services (Hoffman and Novak, 2005). Among them, in-game purchase accounts for a significant proportion (Lin and Sun, 2011). Many game companies, such as DC Universe Online, Tera, and World of Warcraft are eliminating subscription fees for game software, rapidly expanding into “free-to-play” (F2P) online games and basing their revenue on the sale of virtual items and equipment.^1^ Tencent Holdings Ltd, a leading Internet value-added service provider in China, announced total revenue for 2022 at 554.6 billion yuan, where virtual game item sales have contributed a lot to this revenue growth.^2^ We can see that selling virtual goods is becoming more central to the business model of digital games.

In-game items can be divided into two categories: functional items and decorative items. Functional game items increase game users' playing abilities and core competencies; nevertheless, decorative game items allow users to enhance the appearance of in-game items or the game environment, etc. (Hamari, 2015; Bae et al., 2019). Our research considers the multiplayer online battle arena (MOBA) games, which are one of the most popular competitive games today (Thavamuni et al., 2023). Items in MOBA games do not affect fairness, that is, decorative game items because the loss of fairness in this type of game will make players unable to play. In contrast, the key to success in some role-playing games (RPG) is not the player's ability to fight, but the purchase of power-ups to satisfy the player's desire to win, which affects some fairness, that is, functional game items.

Free-to-play gamers can be divided into two groups: those who do not pay and those who buy virtual items (Lin and Sun, 2011). We can distinguish them as paying players and non-paying players. Therefore, to balance these two types of consumers, in-game items are provided by game companies not only through purchase but also through grinding, which makes the game more popular. Grinding in games means spending time in mundane, repetitive activities just to receive new abilities or equipment, that is, only valuable in the game itself (Ryall, 2021). For instance, “Honor of Kings” launched by Tencent, is a typical MOBA game that attracts more than 80 million daily active players and 200 million monthly active players (Yang et al., 2022). One of the highlights is the in-game skins, which refer to the appearance of characters. However, we observe that some characters only have the paid version of skins, while others have both the paid version skins and the grinding version skins. The strategy of the character's skin that the firm provides a grinding version of items helps the game get more attention and buzz. The time needed to obtain the grinding version item and the quality of versions are set by the game company before the version is officially launched. They have a significant influence on players' experience in games and then affect their behavior.

Why do firms offer items in the grinding version when the paid version is profitable? Whether those non-paying players can bring revenue to the firm? Scholars suggest a detailed set of item attributes that drive virtual item purchase decisions, including functional, hedonic, and social attributes (Lehdonvirta, 2009; Wang et al., 2021). Nojima (2007) finds that social motivation is related to virtual identity, and identity leads to higher immersion, thus prompting players to purchase items in the game. As we can notice some rare items are being sold at high prices, for instance, in “Game for Peace,” an out-of-print vehicle skin was sold at a high price on a trading platform, with a price of up to 6,000 yuan. These in-game items, despite not affecting the winning rate, are often sold at high prices, primarily due to conspicuous psychology. Conspicuous consumption primarily emphasizes visual display or public use in front of others (O'cass and McEwen, 2004; Areiza-Padilla and Manzi Puertas, 2021), consumers with conspicuous features often pursue uniqueness, hence their purchasing behavior is often influenced by differentiation. Extensive empirical evidence further suggests that increasing the magnitude of character appearance differences exerts a positive impact on in-game item purchasing behavior (Wagner et al., 2014; Koch and Benlian, 2017; Wang et al., 2023). As a result, game companies can take advantage of people's growing demand for scarcity and set different levels of game items (such as the appearance of virtual items) for consumers to use. Additionally, the more non-paying players a game has or the more time invested by them can contribute to the game's popularity, attracting new players and generating additional revenue for the game company. This revenue may come from sources such as advertising revenue and direct income from converting non-paying players to paying players. Therefore, our study considers the impact of externality.

This study investigates the following interesting and important questions: (1) How does conspicuous consumption affect game companies' product design and pricing? (2) Under what conditions should game companies offer a grinding version of items? (3) If a dual-version of game items is introduced, how do game companies design the time required to obtain grinding version items and the quality differences between in-game items?

The structure of this study is as follows. The related literature is reviewed in Section 2. Section 3 presents the model setup and derives and analyzes the optimal solutions of the two strategies (single-version and dual-version). In Section 4, comparative and numerical studies are conducted to compare two strategies and then obtain more results and managerial implications. Finally, the study presents our conclusions in Section 5. Most of the derivations and detailed discussions are provided in the Appendix.

## 2 Literature review

Our study belongs to the extensive research of virtual goods, and we consider customer behavior with conspicuous features. Thus, the problems examined in this study are related to conspicuous consumption and the pricing decisions of game companies.

### 2.1 Conspicuous consumption

Conspicuous consumption is often characterized by the number of goods sold increasing with the price, which is known as the Veblen effect. Veblen (1899) proposes that it is a consumption activity in which people show their wealth to the public, seek a certain identity, or highlight social status through ultra-practical or wasteful consumption. Since then, an increasing body of literature on conspicuous consumption.

Many scholars focus on the factors that influence conspicuous consumption, such as subjective socioeconomic status (Wang et al., 2022b), non-material social comparison (Zheng et al., 2018), and narcissism (Neave et al., 2020; Sedikides and Hart, 2022). Wang and Tian (2022) revealed the deeper connection between face culture (the composite and unified expression of honor, dignity, self-worth, and prestige given by others and felt by the self in social situations) and conspicuous consumption. Wang et al. (2022a) showed that moral, cognitive, and interpersonal self-uncertainty contribute to a stronger tendency to engage in conspicuous consumption. People are more likely to shift intention when they perceive social pressure from their surroundings (Liu et al., 2023); therefore, the external environment also greatly affects people's tendency toward conspicuous consumption.

Existing studies concentrate on investigating how the firm's pricing decisions are influenced by conspicuous consumption. For instance, Tereyağoğlu and Veeraraghavan (2012) used a rational expectations framework to study the pricing and production decisions of firms facing highly uncertain market demand, strategic consumer behavior, and conspicuous consumption behavior. Zhou et al. (2018) evaluated the firm's pricing and production decisions on conspicuous consumers in the presence of discount sensitivity behavior. Zhu et al. (2022) assumed that conspicuous consumers are less sensitive to price than non-conspicuous consumers, and they developed a game model involving a monopoly firm selling conspicuous products and these two types of consumers and studied how conspicuous consumption affects firm pricing, quality decisions, and revenue. Rao and Schaefer (2013) considered the consumer's pursuit of the intrinsic quality and status effect of durable goods and studied the pricing and product management decisions in the market for conspicuous durable goods. Li (2019) examined how firms selling status goods make vertical line extension decisions when they take consumers' status preferences into account. Wei and Li (2020) investigated how conspicuous behavior and concerns of stock availability influence a luxury firm's operational decisions.

The existing research and models on physical goods cannot effectively capture the features of virtual goods. Some research explores the pricing decisions of virtual goods. Liu et al. (2011) studied the optimal pricing strategy of spreadsheet software products under the influence of piracy and word-of-mouth. Hao and Fan (2014) developed a game theory model to study the pricing of e-books and e-readers under both wholesale and agency pricing models. Chen et al. (2020) priced and designed loot boxes to maximize revenue for video game companies. However, these study does not take into account the psychology of conspicuous consumers. Therefore, we develope this analytical model, and our findings and implications contribute to a better understanding of the conspicuous consumption of virtual goods.

### 2.2 Pricing strategy of game company

Many researchers explored the pricing strategy of game companies, for instance, Lee et al. (2006) proposed a basic strategy that tries to earn revenue by developing premium goods or services to market created by their free goods or services and then they analyzed pricing and service quality strategies for e-business companies providing information services to customers. Civelek et al. (2018) established a Stackelberg game model to discuss the game provider's determination of the optimal pricing of virtual goods and designing game challenge level for free-to-play mobile games in the presence of heterogeneous players and copycat competitors. Chen et al. (2021) considered the snobbery of players and analyzed a mixed revenue model to investigate the effect of introducing advertising incentives to the premium subscription.

Some research focuses on the freemium model. Freemium is a combination of the words “free” and “premium.” It is a business model that offers services or products for free to attract users and then charges for premium features or products (Liu et al., 2014; Hsiao and Chen, 2016). Offering a free trial version and a paid version of the software, as well as setting up in-game paid items in F2P games, are both kinds of the freemium model. Some scholars have studied the former. For example, Haruvy and Prasad (2001) modeled the effect of two decision variables price and freeware quality on the adoption of software using static and evolutionary game theory. Cheng and Tang (2010) examined the trade-off between network effects and the cannibalization effect and aimed to disclose the circumstances under which companies should introduce the free trial product. Geng and Chen (2019) established a model to facilitate this trade-off between increasing the total user volume and maintaining scarcity by considering a reference-based utility shift associated with conspicuous consumption, offering the optimal pricing strategy for a monopoly firm, given different levels of snobbery. Feng et al. (2022) discussed the influence of the positive network externality and the negative security externality on software vendors' optimal freemium versioning strategy and a subsequent security-patching strategy.

Our study concentrates on the pricing of premium items within F2P games. It analyzes the impact of in-game mechanics on game companies, such as the time needed to acquire the grinding version item and the quality differences among various versions. In addition, this study aims to provide insights into the version strategy and pricing decisions of the game company facing consumers with conspicuous consumption psychology.

## 3 Methodology

### 3.1 Model setup and assumptions

The monopoly game company offers a paid version of a specific item (such as a character's skin) with price p∈P=[0,+∞) and quality S∈S=(0,+∞), where the quality of the item refers to its gorgeous degree. This study considers the decorative item and not the functional item; that is, whatever *S* is, it does not affect fairness. Noting that the decorative item that is bought in the game are only valuable in the game itself. We assume that the marginal cost of the firm is zero and the reason is once virtual goods are developed, they are available in unlimited quantities, and the cost of reproduction of digital content is low or close to zero (Shapiro et al., 1999; Na et al., 2017). We assume that the perceived value of the item is heterogeneous among players. θ denotes the preference for quality of the in-game items by an individual player, where θ is uniformly distributed over [0, 1] (Feng et al., 2022). Without the loss of generality, the market is normalized to 1. Conspicuous consumers aspire to be distinct from all other consumers (Arifoğlu et al., 2020; Zhu et al., 2022), so the utility is that a consumer with conspicuous psychology not only relies on the basic valuation of the item but also on the user base. The social value decreases linearly with the quantity of consumers using the product. Similar to Geng and Chen (2019), Wei and Li (2020), and Zhu et al. (2022), we denote α > 0 as the conspicuous intensity, which is homogeneous among consumers. It is crucial for game companies as it helps them determine version strategy and adjust optimal prices to maximize their revenue. All notations are summarized in Table 1.

**Table 1:** Notations.

**Notation**	**Explanation**
Sets and indices	
*i* ∈ {*H, L*}	The type of in-game items, i.e., H for the paid version item and L for the grinding version item
Variables	
*p*	Price of the paid version item (*p* ≥ 0)
*Q*_*i*_	The quantity of players who get the i-type item (*Q*_*i*_ ≥ 0)
*π*	The revenue of the game company
Parameters	
θ	Consumer type, i.e., consumer's preference for the quality of in-game items (θ is uniformly distributed over [0, 1])
*t*	Time needed to obtain the grinding version item (*t* ≥ 0)
*S*	The quality of the paid version item (*S* > 0)
Δ	The quality difference between two versions (0 < Δ < *S*)
α	The conspicuous intensity of the customer (α ≥ 0)
λ	The cross-quantity sensitivity coefficient (λ ≥ 0)
η	Time cost coefficient (0 < η < 1)
μ	The coefficient of externality revenue (η < μ < 1)

### 3.2 Single-version

First, we considered that the game company does not introduce the grinding version that only offers the paid version item to consumers. In this case, the consumer's valuation for the quality of the paid version item is represented by θ*S*, and we used −α*Q*_*H*_ to represent the conspicuous utility, where *Q*_*H*_ is the quantity of customers who purchase the item. It implies that the more buyers there are, the less show-off the customer feels. Thus, the utility of the consumer who buys the item with the preference θ can be expressed as


(1) $$\begin{array}{l} u_{paid}=\theta S-p-\alpha Q_{H} \end{array}$$

**Figure 1:**
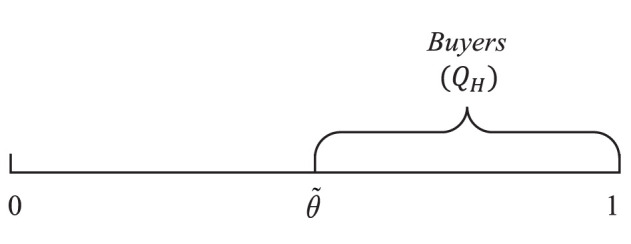
Demand under the single-version strategy.

Customers buy the item if and only if Eq. 1 ≥ 0; that is, θ is larger than or equal to a threshold $\tilde{\theta }$, where $\tilde{\theta }=\frac{\alpha Q_{H}+p}{S}$. Since θ is uniformly distributed within the interval [0, 1], thus, from $\tilde{\theta }$ to 1 is the quantity of buyers, as shown in Figure 1. Therefore, the quantity of buyers is indicated by $Q_{H}=1-\tilde{\theta }$. Thus, we can get the demand of the item as


(2) $$\begin{array}{l} Q_{H}=\frac{S-p}{\alpha +S} \end{array}$$

The firm seeks to maximize the revenue


(3) $$\begin{array}{l} \underset{p>0}{\text{Max}}\pi =pQ_{H} \end{array}$$

Proposition 1. In the case of single-version strategy:

(1) The optimal price and the corresponding demand of the paid version item are $p^{*}=\frac{1}{2}S$, $Q_{H}{\;}^{*}=\frac{S}{2\left(\alpha +S\right)}$. The maximum revenue of the firm is $\pi^{*}=\frac{S^{2}}{4\left(\alpha +S\right)}$.

(2) $\frac{\partial p^{*}}{\partial S}>0$, $\frac{\partial Q_{H}{\;}^{*}}{\partial S}>0$, $\frac{\partial \pi^{*}}{\partial S}>0$, $\frac{\partial p^{*}}{\partial \alpha }=0$, $\frac{\partial Q_{H}{\;}^{*}}{\partial \alpha }<0$, and $\frac{\partial \pi^{*}}{\partial \alpha }<0$.

Proposition 1 shows the optimal price, and the corresponding demand and revenue, and they all increase with the quality of the version because the consumer's utility increases with the quality of the paid version of the item. However, the optimal price does not change with α, the demand and the revenue decrease with the conspicuous intensity. If players do not have conspicuous psychology, the utility is not affected by the behavior of other buyers. Because of the psychology of showing off, the higher the sales, the greater the negative effect on utility, thus consumers' willingness to pay decreases, and the higher the conspicuous intensity is, the greater the impact of sales on consumers' utility, leading to a decline in the quantity of buyers, which in turn is detrimental to the revenue.

### 3.3 Dual-version

In this subsection, we consider the scenario under which the game company not only provides the paid version item but also a relatively low-quality version, which can be obtained by grinding. We assume that the time needed to get the grinding version is *t*, and the consumers' time cost coefficient is η. The quality of the grinding version item is *S* − Δ, where 0 < Δ < *S*. Note that the quality gap Δ between two versions of the in-game item also represents the difference in the gorgeous degree. Then, the consumer valuation for the quality of the paid version and the grinding version item can be expressed by θ*S* and θ(*S* − Δ), respectively.

How players choose is based on the utility they can obtain from the two versions. Due to the conspicuousness, we can see that the utility of buyers, who purchase the paid version item, is not only related to the quantity of buyers *Q*_*H*_ but also the quantity of grinding version users *Q*_*L*_. The conspicuous term of utility can be indicated by −α(*Q*_*H*_ − λ*Q*_*L*_), where the cross-quantity sensitivity coefficient λ ≥ 0 means the extent to which the quantity of grinding version users affects conspicuous buyers, α is the intensity of conspicuousness. That means the utility from the show-off psychology decreases as the quantity of buyers increases but increases as the quantity of grinding version users increases. Therefore, the utility of a player, if he chooses the paid version item, is


(4) $$\begin{array}{l} u_{paid}=\theta S-p-\alpha \left(Q_{H}-\lambda Q_{L}\right) \end{array}$$

and the utility of a player if he chooses the grinding version item is


(5) $$\begin{array}{l} u_{grinding}=\theta \left(S-\Delta \right)-\eta t \end{array}$$

**Figure 2:**
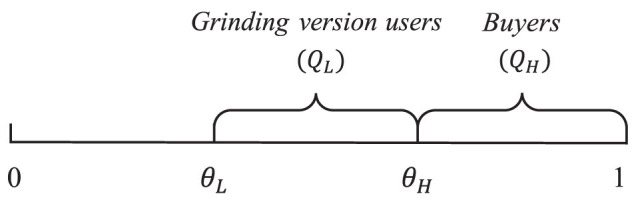
Demands under the dual-version strategy.

Consumers with a preference for quality θ will choose the grinding version item if their utility is larger than or equal to zero and larger than buying the item. Two marginal values of preference of quality are denoted by θ_*L*_ and θ_*H*_. Consumers with preference θ_*L*_ are indifferent between the grinding version item and not obtaining the item, that is, Eq. 5 = 0, while consumers with preference θ_*H*_ are indifferent between buying the item and obtaining the grinding version item, that is, Eq. 4 = 0. Figure 2 shows the demands of two versions. Accordingly, the sizes of the user base of the grinding version and the paid version are


(6) $$\begin{array}{l} Q_{L}=\frac{\left(\alpha +p\right)\left(S-\Delta \right)-\eta t\left(\alpha +S\right)}{\left(S-\Delta \right)\left(\alpha \lambda +\alpha +\Delta \right)} \end{array}$$

(7) $$\begin{array}{l} Q_{H}=\frac{\left(S-\Delta \right)\left(\Delta -p+\eta t\right)+\alpha \lambda \left(S-\Delta -\eta t\right)}{\left(S-\Delta \right)\left(\alpha \lambda +\alpha +\Delta \right)} \end{array}$$

We need to ensure that there are players who are willing to spend time to obtain the item in the market, that is, *Q*_*L*_ ≥ 0, hence, according to Eq. 6, we can conclude that


(8) $$\begin{array}{l} p\geq \max\left(\frac{\eta t\left(\alpha +S\right)}{S-\Delta }-\alpha ,0\right)=P_{1} \end{array}$$

In other words, if the price of the paid version item is extremely low, there are only players who buy the paid version item and those who do not. That is, potential consumers with net utility greater than 0 will choose to buy the paid version item, which makes the game company offer the grinding version of the strategic product ineffective.

Similarly, to avoid triviality and ensure some players are willing to buy the item in the market, we assume that *Q*_*H*_ ≥ 0. According to Eq. 7, we can conclude that


(9) $$\begin{array}{l} 0\leq p\leq \frac{\alpha \lambda \left(\Delta -S+\eta t\right)}{\Delta -S}+\Delta +\eta t=P_{2} \end{array}$$

Noting that we only consider the situation that *P*_2_ > *P*_1_, which is equivalent to $0\leq t<\frac{S-\Delta }{\eta }$. Equations 8, 9 also imply that the cutoff price of the paid version item increases with the time needed to obtain the grinding version item.

We know that the purchasing behavior of paying players in the game can bring benefits to the company. However, non-paying players can also indirectly bring benefits to the company. The externality is defined as the indirect income or costs generated for other economic participants by the production and consumption of goods (Raz and Ovchinnikov, 2015; Chen et al., 2019; Xie et al., 2021). By considering positive externality, we depict the externality revenue generated by grinding version users here with μ*tQ*_*L*_, where $\eta <\mu <\frac{S}{S-\Delta }\eta$ means the coefficient of externality revenue is greater than the time cost coefficient but falls below a specific threshold. Therefore, the revenue of the firm can be expressed as follows:


(10) $$\begin{array}{l} \underset{P_{1}\leq p\leq P_{2}}{\text{Max}}\pi =pQ_{H}+\mu tQ_{L} \end{array}$$

Solving the above optimization problem, we can obtain the optimal price of the paid version item when the company provides two versions in Proposition 2.

Proposition 2. The optimal price of the paid version item under the dual-version strategy is


(11) $$\begin{array}{l} p^{*}=\{\begin{array}{cc} \frac{-X+S(Y+Z)-\mu t\Delta }{2(S-\Delta )} & 0\leq t<L_{t}\\ \frac{\eta t(\alpha +S)}{S-\Delta }-\alpha & L_{t}\leq t<\frac{S-\Delta }{\eta } \end{array} \end{array}$$

where *X* = (αλ+Δ)(Δ+η*t*), *Y* = Δ+η*t*, *Z* = αλ+μ*t*, *L*_*t*_ = $\frac{X\left(S-\Delta \right)}{\alpha \eta \left(\lambda +2\right)+\Delta \left(\eta +\mu \right)+S\left(\eta -\mu \right)}$.

Corollary 1. If the company adopts the optimal price, the quantity of grinding version users is


(12) $$\begin{array}{l} Q_{L}{\;}^{*}=\{\begin{array}{ll} \frac{-NY+S(Z+2\alpha +\Delta -\eta t)-\mu t\Delta }{M} & 0\leq t<L_{t}\\ 0 & L_{t}\leq t<\frac{S-\Delta }{\eta } \end{array} \end{array}$$

where *M* = 2(*S* − Δ)(αλ + α + Δ), *N* = α(λ + 2) + Δ.

The quantity of buyers is


(13) $$\begin{array}{l} Q_{H}{\;}^{*}=\{\begin{array}{ll} \frac{-X+S(Y+\alpha \lambda -\mu t)+\mu t\Delta }{M} & 0\leq t<L_{t}\\ 1-\frac{\eta t}{S-\Delta } & L_{t}\leq t<\frac{S-\Delta }{\eta } \end{array} \end{array}$$

The maximum revenue under the dual-version strategy of the company is


(14) $$\begin{array}{l} \pi^{*}=\{\begin{array}{ll} \begin{array}{l} \frac{1}{2M(S-\Delta )}({\left(X-S(Y+\alpha \lambda )\right)}^{2}\\ -\mu^{2}t^{2}{\left(S-\Delta \right)}^{2}+2\mu t(S-\Delta )\\ \left(-NY+S(N+t(\mu -\eta ))-\mu t\Delta )\right) \end{array} & 0\leq t<L_{t}\\ \frac{\left(S-Y)(\eta t(\alpha +S)-\alpha (S-\Delta )\right)}{{\left(S-\Delta \right)}^{2}} & L_{t}\leq t<\frac{S-\Delta }{\eta } \end{array} \end{array}$$

Proposition 2 shows under what circumstances the company induces customers to choose the grinding version. When the time needed to acquire the grinding version item *t* is shorter than the threshold *L*_*t*_, the company has the incentive to allow both versions of items to coexist in the market. That means the introduction of the grinding version decreases the quantity of buyers but increases the quantity of grinding version users, which leads to a higher utility for buyers due to the psychology of conspicuousness. However, when *t* is larger than or equal to *L*_*t*_, the company sets a price to induce all players to either choose the paid version items or not use items at all.

In the following, we analyze how the optimal price and the corresponding demands ($Q_{L}{\;}^{*}$ and $Q_{H}{\;}^{*}$) change with parameters *t* and α.

Proposition 3. In the case of dual-version strategy, p^*^, $Q_{L}{\;}^{*}$, and $Q_{H}{\;}^{*}$ have the following properties with *t*:

(1) If *t* ∈ [0, *L*_*t*_), $\frac{\partial Q_{L}{\;}^{*}}{\partial t}<0$, $\frac{\partial Q_{H}{\;}^{*}}{\partial t}<0$. Furthermore, $\frac{\partial p^{*}}{\partial t}>0$ when $0\leq \lambda <\frac{\left(\eta +\mu \right)\left(S-\Delta \right)}{\eta \alpha }$, $\frac{\partial p^{*}}{\partial t}<0$ when $\lambda \geq \frac{\left(\eta +\mu \right)\left(S-\Delta \right)}{\eta \alpha }$.

(2) If $t\in \left[L_{t},\frac{S-\Delta }{\eta }\right)$, $Q_{L}{\;}^{*}=0$, $\frac{\partial Q_{H}{\;}^{*}}{\partial t}<0$, $\frac{\partial p^{*}}{\partial t}>0$.

Proposition 3(1) depicts that when *t* is lower than the threshold *L*_*t*_, as the longer time needed to obtain the grinding version item, the quantity of such non-paying players $Q_{L}{\;}^{*}$ decreases and then weakens the showoff of buyers which lead to the reduction of the quantity of such buyers $Q_{H}{\;}^{*}$.

When λ is lower than a threshold $\frac{\left(\eta +\mu \right)\left(S-\Delta \right)}{\eta \alpha }$, the reduction of $Q_{H}{\;}^{*}$ causes the utility of buyers increases, and the optimal price of the paid version item increases with the time needed to obtain the grinding version item. However, when λ is greater than or equal to this threshold, the gradual reduction of $Q_{L}{\;}^{*}$ results in the utility of buyers decreasing, thus dragging down the price of the paid version item.

Proposition 3(2) indicates that when *t* is larger or equal to *L*_*t*_, the demand of the grinding version is 0, which leads to a negative effect on the conspicuousness of buyers; thus, the quantity of buyer reduces with *t*, and the utility of buyers increases. So, the firm has the motivation to raise the optimal price constantly.

Proposition 4. In the case of dual-version strategy, p^*^, $Q_{L}{\;}^{*}$, and $Q_{H}{\;}^{*}$ have the following properties with α when $\frac{\Delta \left(S-\Delta \right)}{\Delta \left(\eta +\mu \right)+S\left(\eta -\mu \right)}\leq t<\frac{S-\Delta }{\eta }$:

(1) If α ∈ [0, *L*_α_), where $L_{\alpha }=\frac{\Delta \left(\mu t+Y\right)-S\left(\Delta +t\left(\mu -\eta \right)\right)}{\left(\lambda +2\right)\left(S-Y\right)}$, $Q_{L}{\;}^{*}=0$ and $\frac{\partial Q_{H}{\;}^{*}}{\partial \alpha }=0$, $\frac{\partial p^{*}}{\partial \alpha }<0$.

(2) If α ∈ [*L*_α_, +∞), $\frac{\partial Q_{L}{\;}^{*}}{\partial \alpha }>0$ and $\frac{\partial Q_{H}{\;}^{*}}{\partial \alpha }<0$, $\frac{\partial p^{*}}{\partial \alpha }>0$.

Proposition 4 states that the optimal price of the paid version item p^*^ decreases first and then increases with the conspicuous intensity α, and this conclusion is consistent with the finding of Geng and Chen (2019). The demand of the grinding version $Q_{L}{\;}^{*}$ first remains unchanged and then increases, and the demand of the paid version $Q_{H}{\;}^{*}$ first remains unchanged and then decreases.

When α is less than the threshold *L*_α_, $Q_{L}{\;}^{*}$ is equal to zero, buyers' utility decreases with α, which leads to the reduction of the optimal price. While when α is larger than or equal to the threshold *L*_α_, the quantity of the grinding version users increases with α, but the quantity of buyers decreases with α, which leads to the larger utility of buyers, and then the company adjusts the optimal price upward. This reflects a common phenomenon in real life that excessive conspicuous intensity will lead to a continuous price increase.

## 4 Strategies comparison and numerical studies

We have obtained the equilibrium solution results of two strategies and analyzed them, respectively. In this section, we compare the two strategies and further study which strategy is better from the perspective of the company. We use $p_{s}{\;}^{*}$, $Q_{s}{\;}^{*}$, and $\pi_{s}{\;}^{*}$ to denote the optimal price and the corresponding demand and revenue of providing only a single-version respectively; and let $p_{d}{\;}^{*}$ and $\pi_{d}{\;}^{*}$ denote the optimal price and revenue of providing dual-version.

Proposition 5. (1) When $\frac{\Delta \left(S-\Delta \right)}{\Delta \left(\eta +\mu \right)+S\left(\eta -\mu \right)}\leq t<\frac{S-\Delta }{\eta }$, $p_{s}{\;}^{*}<p_{d}{\;}^{*}$.

(2) When $L_{t}\leq t<\frac{S-\Delta }{\eta }$, $Q_{s}{\;}^{*}>Q_{H}{\;}^{*}$ and $\pi_{s}{\;}^{*}>\pi_{d}{\;}^{*}$, $L_{t}\geq \frac{\Delta \left(S-\Delta \right)}{\Delta \left(\eta +\mu \right)+S\left(\eta -\mu \right)}$.

Proposition 5 indicates that when *t* is set in the range $\frac{\Delta \left(S-\Delta \right)}{\Delta \left(\eta +\mu \right)+S\left(\eta -\mu \right)}\leq t<\frac{S-\Delta }{\eta }$, the optimal price under the single-version strategy is always lower than the paid version item that in the dual-version strategy. When $L_{t}\leq t<\frac{S-\Delta }{\eta }$, the demand of the single-version item is higher than the paid version item in the dual-version strategy. Proposition 5(2) further illustrates that when the time needed to obtain the grinding version item *t* is larger than or equal to the threshold *L*_*t*_, nobody chooses to use the grinding version item, and the quantity of buyers under the dual-version strategy is lower than that under the single-version strategy, which resulting in the single-version strategy being more profitable.

Then, we study the effects of the time needed to obtain the grinding version item (t), the customer's conspicuous intensity (α), and the quality difference between the two versions (Δ) as well as compare the equilibrium outcomes to obtain the optimal version strategy for the game company. Based on the assumptions established in models, parameter values are assigned as shown in Table 2.

**Table 2:** Parameter values.

**Parameters**	*t*	α	*S*	Δ	λ	η	μ
Values	3	2	4	2	0.8	0.5	0.6

### 4.1 Impact of time needed to obtain the grinding version item

**Figure 3:**
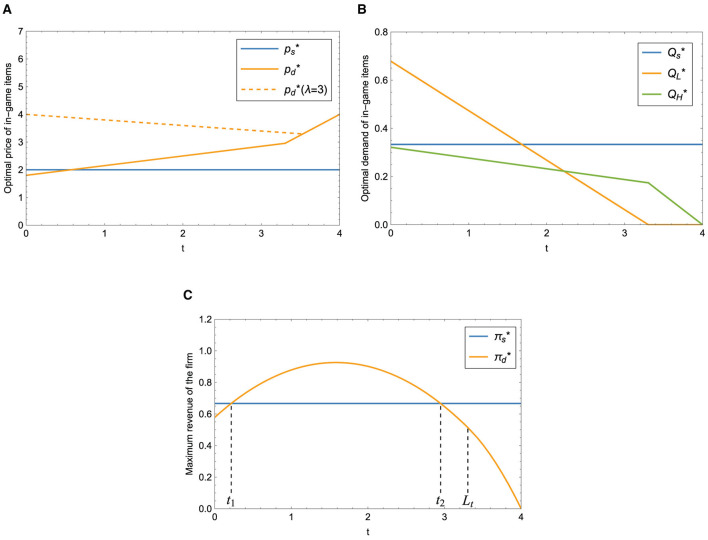
Impact of *t* on different strategies. **(A)** Impact of *t* on optimal price. **(B)** Impact of *t* on optimal demand. **(C)** Impact of *t* on maximum revenue.

According to Proposition 3, in the case of dual-version strategy, we can see that when λ is lower than a threshold, the optimal price of the paid version increases with *t*. Otherwise, the optimal price decreases first and then increases with *t*, as shown in Figure 3A. The optimal price of the single version is not affected by *t*. It can be observed from Figure 3B that the demand of the grinding version decreases as the time needed to obtain the grinding version item increases, reaching zero when *t* ≥ *L*_*t*_. Simultaneously, the utility of buyers decreases as the quantity of grinding version users declines, resulting in a decrease in the demand of the paid version as well.

Figure 3C illustrates the maximum revenue of the firm under the single-version (the blue curve) and the dual-version (the orange curve) strategy respectively. It is easy to see that the revenue of providing a single version does not vary with the time needed to obtain the grinding version item; when *t*_1_ < *t* < *t*_2_ (*t*_1_, *t*_2_ are two thresholds), it is more profitable to provide dual-version items; otherwise, the single-version strategy is a better choice for the firm. In other words, the company should adjust the time needed to acquire the grinding version item and try to offer dual-version items to increase revenue. Furthermore, we can conclude that the introduction of the grinding version decreases the quantity of buyers but increases the utility of buyers which can improve the optimal price of the paid version item.

### 4.2 Impact of conspicuous intensity

**Figure 4:**
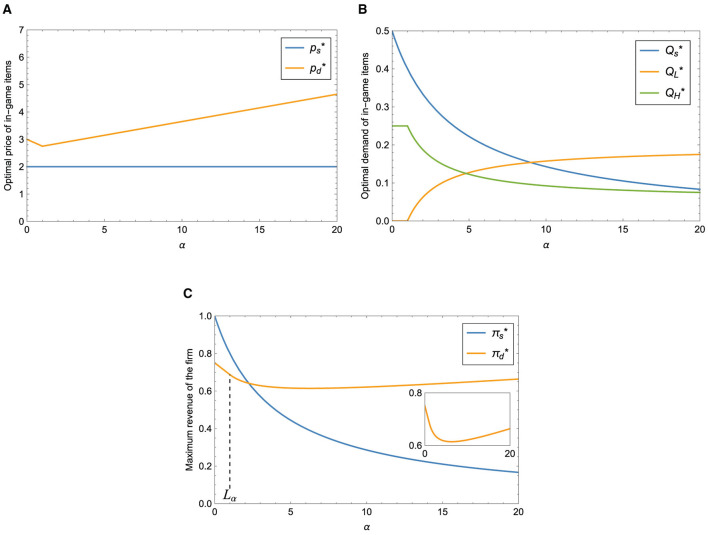
Impact of α on different strategies. **(A)** Impact of α on optimal price. **(B)** Impact of α on optimal demand. **(C)** Impact of α on maximum revenue.

Figure 4A shows that the optimal price of the paid version item under the dual-version strategy first decreases and then increases with conspicuous intensity α. It is always higher than the price of the single-version. On the one hand, when the conspicuous intensity is weak, conspicuous psychology will lead to the reduction of the optimal price of the paid version items because the utility of players who choose the paid version item is not only larger or equal to zero but also larger or equal to the utility of players who choose the grinding version. To improve buyers' utility, the company has to lower the price. On the other hand, when the intensity of conspicuous is higher than or equal to a threshold *L*_α_, the optimal price increases monotonously. That is, the behavior of conspicuousness raises unit prices. At this point, people are more likely to choose the grinding version items, and as a result, many players stop buying items, which can be seen in Figure 4B.

Figure 4C illustrates the maximum revenue of both single-version and dual-version. As the conspicuous intensity increases, the revenue of the dual-version strategy first declines and then shows an increasing trend. We can see that the price of the item under the single-version strategy remains constant, and the corresponding demand declines, so the firm's maximum revenue from offering a single version falls. The maximum revenue of the single-version strategy is first higher than that obtained by offering dual versions, and then it is exceeded by the dual-version strategy. Under this set of parameters, we suggest that when the conspicuous intensity is not that strong, it is more profitable for the company to adopt the single-version strategy; otherwise, the company should implement the dual-version strategy to increase its revenue.

### 4.3 Impact of quality difference

The quality difference distinguishes the paid version from the grinding version. In the following, to expand the range of value differences for analysis, we increase the quality *S* of the paid version item to 10. The results can give suggestions on how to control the quality difference.

**Figure 5:**
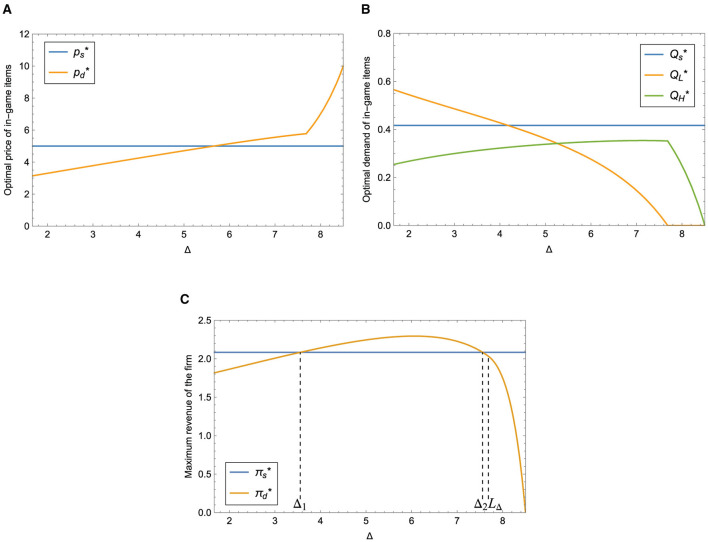
Impact of Δ on different strategies. **(A)** Impact of Δ on optimal price. **(B)** Impact of Δ on optimal demand. **(C)** Impact of Δ on maximum revenue.

Similarly, we can also obtain the effect of the quality difference between the two versions on optimal strategy and pricing. The critical value *L*_Δ_ and the maximum revenue expression are shown in the Appendix. The comparison of the two strategies under the influence of quality difference is shown in Figure 5. Obviously, the single version item has no quality difference, so its equilibrium solutions do not change with Δ. Figure 5A shows that the optimal price of dual-version increases with the quality difference. As shown in Figure 5B, when the quality difference is lower than the threshold *L*_Δ_, the demand of the paid version decreases, but the demand of the grinding version increases; while when Δ is larger than or equal to *L*_Δ_, the quantity of grinding version users is 0 and buyers drop to 0, which lead to the company's revenue plummet. It can be seen from Figure 5C that the maximum revenue of providing dual-version items is lower than that of providing the single version item at first and then exceeds the single version, reaching the highest point and then decreasing. When the quality difference is within a certain range (Δ_1_ < Δ < Δ_2_), it is more profitable to provide dual-version items. Therefore, to maximize the revenue, the game company can try to adopt a dual-version strategy and choose a moderate level of difference.

### 4.4 Optimal version strategy

**Figure 6:**
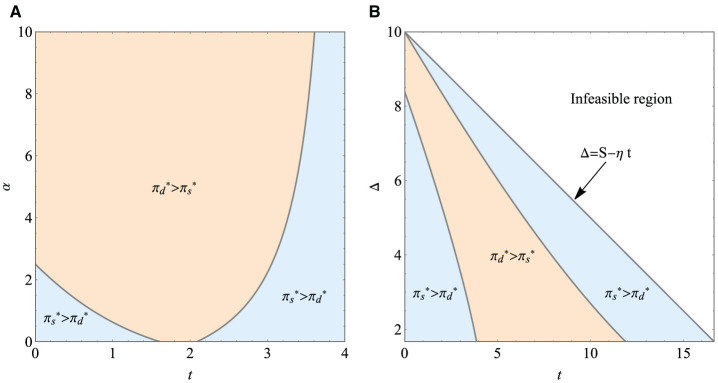
Firm's optimal strategy (revenue comparison). **(A)** Optimal strategy with the changing of *t* and α. **(B)** Optimal strategy with the changing of *t* and Δ.

The optimal strategy for the items' version provided by the game company is shown in Figure 6. Figure 6A shows that when conspicuous intensity and the time needed to obtain the grinding version are both at low level, or when the time needed to obtain the grinding version is extended, it is more profitable for the company to offer single-version. Otherwise, it is more profitable to offer dual-version.

Figure 6B shows the impact of the time needed to obtain the grinding version item and the quality difference on the firm's revenue under two different strategies. We can get the following findings. First, if there is a small difference between the two versions, and the time needed to obtain the grinding version is too long or too short, the single-version strategy is more profitable than the dual-version strategy. Second, when the time needed to obtain the grinding version item is short, and the version difference is too large or does not exceed a certain threshold, the single-version strategy is a better option for the game company. Finally, when the time required to obtain the grinding version item exceeds a certain threshold, no matter what the version difference is, the single-version strategy is always better than the dual-version strategy. If and only if the time needed to obtain the grinding version item and the difference between the two versions are at moderate level, the company will be able to make more profitable by adopting the dual-version strategy. It is worth noting that we compare the impact of these two parameters on the revenue satisfying the previously mentioned constraint $S-\frac{\eta S}{\mu }<\Delta <S-\eta t$.

## 5 Discussion

More and more game companies are innovating the in-game purchase mechanism. Different levels of items can stimulate the conspicuous psychology of consumers and make the paid version item more attractive. We developed two analytical models considering conspicuous consumption, product quality differences, and the time needed to obtain the grinding version item, capture the optimal pricing of virtual goods in the presence of conspicuous consumers, and analyze how the firm chooses the optimal strategy. The results of our study are as follows.

First, we suggest that when a dual-version strategy is provided, to allow users of both versions to coexist in the market, the optimal price should be set within a certain range. When the time needed to obtain the grinding version item is lower than a certain threshold, the company shall set the price of the paid version item at the optimal solution under unconstrained conditions; otherwise, the company sets a price to induce all players to either choose the paid version items or not use items at all. That means the company does not introduce a grinding version.

Second, we propose that game companies offer dual-version items rather than a single version when players' conspicuous intensity is high. Because in the case of strong conspicuous intensity, as the conspicuous intensity increases, the item's price of the paid version in the dual-version strategy continues to increase, and the firm can obtain better revenue from this than from the single version.

Furthermore, the time needed to obtain the grinding version item is an important factor in the company's profitability. We find that the longer the time needed to obtain the grinding version item, the corresponding number of non-paying players decreased, and the number of paying players also decreased due to conspicuous psychology. Our analytical results also show the game company should adjust the time needed to obtain the grinding version item and widen the gap between versions appropriately, trying to provide two versions of items, which is conducive to the revenue growth of the company.

To the best of our knowledge, our study is the first attempt to depict the popularity of virtual products that bring indirect revenue into the model. However, there are some limitations in this study due to our model assumptions, and subsequent work can be extended and improved in the following directions. In the current study, we have not taken into account the impact of the network externality on the potential expansion of the market size, as Cheng and Tang (2010) and Geng and Chen (2019) have done. In addition, subsequent research can consider the random market demand and explore game suppliers' product strategy and pricing decisions under competition. Finally, future research can also be designed to conduct experiments or employ actual data to verify the results generated from the models.

## Data availability statement

The raw data supporting the conclusions of this article will be made available by the authors, without undue reservation.

## Author contributions

FL: Conceptualization, Methodology, Writing – review & editing, Validation, Funding acquisition. JC: Visualization, Formal analysis, Writing – original draft, Validation. TX: Software, Visualization, Supervision, Writing – review & editing.

## Footnotes

^1^ https://venturebeat.com/games/the-cost-of-failing-at-freemium/

^2^ https://static.www.tencent.com/uploads/2023/03/22/8d304603bd688ad947575f738ce62fca.pdf

## Appendix

### Marginal values of preference of quality in dual-version

In section 3.3, we discuss two marginal values of preference of quality θ_*L*_ and θ_*H*_. They satisfy the following two equations.

$$\theta_{L}=\frac{\eta t}{S-\Delta }$$

$$\theta_{H}=\frac{\alpha Q_{H}-\alpha \lambda Q_{L}+P-\eta t}{\Delta }$$

Because *Q*_*L*_ = θ_*H*_ − θ_*L*_, *Q*_*H*_ = 1 − θ_*H*_, we have

$$\theta_{L}=\frac{\eta t}{S-\Delta }$$

$$\theta_{H}=\frac{\left(S-\Delta \right)\left(\alpha +P-\eta t\right)+\alpha \eta \lambda t}{\left(S-\Delta \right)\left(\alpha \lambda +\alpha +\Delta \right)}$$

### Proof of Proposision 2

By checking the optimal condition for the revenue function, we can find that $\frac{d^{2}\pi }{dp^{2}}<0$, it shows that π is a strictly concave function in *p*, and hence, there exists a maximum of π, and then we can get the unconstrained equilibrium $P_{0}=\frac{-X+S\left(Y+Z\right)-\mu t\Delta }{2\left(S-\Delta \right)}$. After that, we need to judge the magnitude of *P*_0_, *P*_1_, and *P*_2_ under the conditions we assumed. Hence, we have the proposition.

### Proof of Proposision 3

When $L_{t}\leq t<\frac{S-\Delta }{\eta }$, the optimal price is the boundary *P*_1_, and $\frac{\partial p^{*}}{\partial t}>0$. While when 0 ≤ *t*<*L*_*t*_, the optimal price is the unconstrained equilibrium solution *P*_0_, and $\frac{\partial p^{*}}{\partial t}>0$ if $0\leq \lambda <\frac{\left(\eta +\mu \right)\left(S-\Delta \right)}{\eta \alpha }$; however, $\frac{\partial p^{*}}{\partial t}<0$ if $\lambda \geq \frac{\left(\eta +\mu \right)\left(S-\Delta \right)}{\eta \alpha }$.

### Proof of Proposision 4

We explore the effect of conspicuous intensity on the optimal value. If α ≥ *L*_α_, the optimal value can always be achieved under unconstrained conditions. Otherwise, the optimal value is the boundary *P*_1_ that needs to be satisfied when *Q*_*L*_ is larger than or equal to 0. Therefore, we can then rewrite the revenue function into

$$\begin{array}{c} \pi^{*}=\{\begin{array}{ll} \begin{array}{l} \frac{1}{2M(S-\Delta )}({\left(X-S(Y+\alpha \lambda )\right)}^{2}-\mu^{2}t^{2}{\left(S-\Delta \right)}^{2}\\ +2\mu t(S-\Delta )(-NY+S(N+t(\mu -\eta ))-\mu t\Delta )) \end{array} & \alpha \geq L_{\alpha }\\ \frac{\left(S-Y)(\eta t(\alpha +S)-\alpha (S-\Delta )\right)}{{\left(S-\Delta \right)}^{2}} & 0\leq \alpha <L_{\alpha } \end{array} \end{array}$$

### Critical value of quality difference

$$\begin{array}{l} L_{\Delta }=\frac{1}{2}(S-\phi -\varphi )+\frac{1}{2}\sqrt{S^{2}+\phi^{2}+\varphi^{2}+2S(\phi +t(\mu -3\eta ))-2t\phi (\eta -\mu )} \end{array}$$

where ϕ = α(λ+2) and φ = *t*(η+μ).

In Section 4.3, the maximum revenue can be expressed as

$$\begin{array}{c} \pi^{*}=\{\begin{array}{ll} \begin{array}{l} \frac{1}{2M(S-\Delta )}({\left(X-S(Y+\alpha \lambda )\right)}^{2}-\mu^{2}t^{2}{\left(S-\Delta \right)}^{2}\\ +2\mu t(S-\Delta )(-NY+S(N+t(\mu -\eta ))-\mu t\Delta )) \end{array} & S-\frac{\eta S}{\mu }<\Delta <L_{\Delta }\\ \frac{\left(S-Y)(\eta t(\alpha +S)-\alpha (S-\Delta )\right)}{{\left(S-\Delta \right)}^{2}} & L_{\Delta }\leq \Delta <S-\eta t \end{array} \end{array}$$
